# Radiological Assessment of Fusobacterium Necrophorum Osteomyelitis and Septic Arthritis: A Case Report

**DOI:** 10.7759/cureus.72264

**Published:** 2024-10-24

**Authors:** Alexis Nguyen, Katherine E Guardado, Arif Musa, Chris Kelly

**Affiliations:** 1 Radiology, Wayne State University School of Medicine, Detroit, USA; 2 Osteopathic Medicine, Michigan State University, Lansing, USA; 3 Diagnostic Radiology, Detroit Medical Center, Wayne State University School of Medicine, Detroit, USA; 4 Radiology, Detroit Medical Center, Wayne State University, Detroit, USA

**Keywords:** debridement, fusobacterium, lemierre's syndrome, magnetic resonance imaging, neck space infections, necrophorum, osteomyelitis, osteonecrosis, pediatrics, septic arthritis

## Abstract

This case report presents a unique case of pediatric Fusobacterium necrophorum osteomyelitis and septic arthritis, emphasizing the role of radiological imaging in diagnosis, treatment planning, and monitoring. Fusobacterium necrophorum, typically a member of the head and neck flora, is an uncommon causative agent of osteomyelitis, making this case particularly noteworthy. A 17-year-old male, with a history of recurrent otalgia, presented with worsening right otalgia, otorrhea for six days, headache, decreased range of motion of the neck to the right, and right hip pain. Radiological assessment, including magnetic resonance imaging (MRI) and other diagnostic modalities, played a pivotal role in characterizing the extent of the disease, guiding surgical interventions, and monitoring treatment response. This case report demonstrates the significance of interdisciplinary collaboration and radiological expertise in managing complex musculoskeletal infections.

## Introduction

Fusobacterium necrophorum, a gram-negative anaerobic rod typically residing within the oropharyngeal flora, is known for its pathogenicity in head and neck infections, including peritonsillar abscesses [1], Lemierre's syndrome [2], and deep neck space infections [3]. Although F. necrophorum is the most common of the Fusobacterium species to cause osteomyelitis [4], it is uncommon for F. necrophorum to manifest as osteomyelitis and septic arthritis in regions far removed from the head and neck [5]. This atypical presentation challenges clinicians and requires a high level of suspicion for an accurate diagnosis. The aim of this case report is to elucidate a rare pediatric case involving F. necrophorum-related osteomyelitis, specifically affecting the hip. F. necrophorum's migration to remote skeletal sites is a phenomenon that remains poorly understood. The report underscores the need for a comprehensive understanding of unusual pathogens and their atypical presentations in pediatric osteomyelitis. In this context, radiological imaging, particularly MRI, assumes a pivotal role in identifying the extent of the disease and guiding surgical interventions [6]. This report emphasizes the significance of interdisciplinary collaboration and radiological expertise in managing complex cases and highlights the importance of considering uncommon pathogens in the assessment and treatment of pediatric patients with osteomyelitis.

## Case presentation

A 17-year-old male, with a history of chronic right otitis media and mastoiditis treated with tympanostomy tubes and antibiotics, presented with worsening right otalgia, otorrhea, headache, non-meningeal neck pain with decreased range of motion, and acute right hip pain, without fever. The initial clinical presentation, including elevated white blood cell count, elevated erythrocyte sedimentation rate and c-reactive protein, physical examination (point tenderness and decreased range of motion), no traumatic etiology, and negative hip radiography, raised concerns of possible osteomyelitis in the right hip and worsening chronic otitis media/mastoiditis. Radiological assessment, including MRI, was performed to evaluate the extent of the disease.

**Figure 1:**
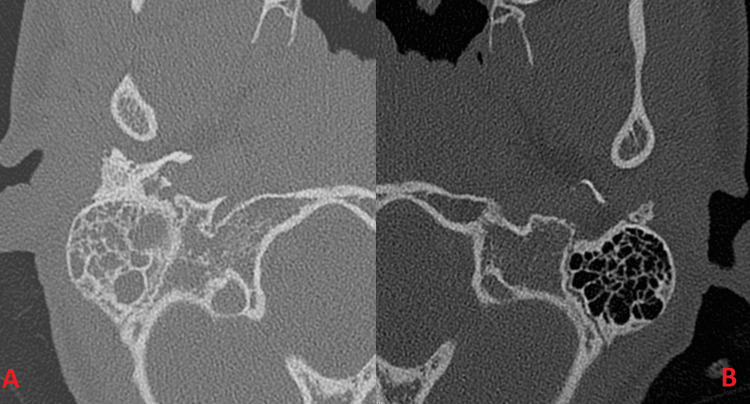
Axial CT head with IV contrast at the level of the mastoid air cells and posterior fossa. The right (A) and left (B) mastoid air cells are pictured. There is opacification of the right mastoid air cells, middle ear, and external auditory canal, which likely represents acute otomastoiditis and otitis externa.

**Figure 2:**
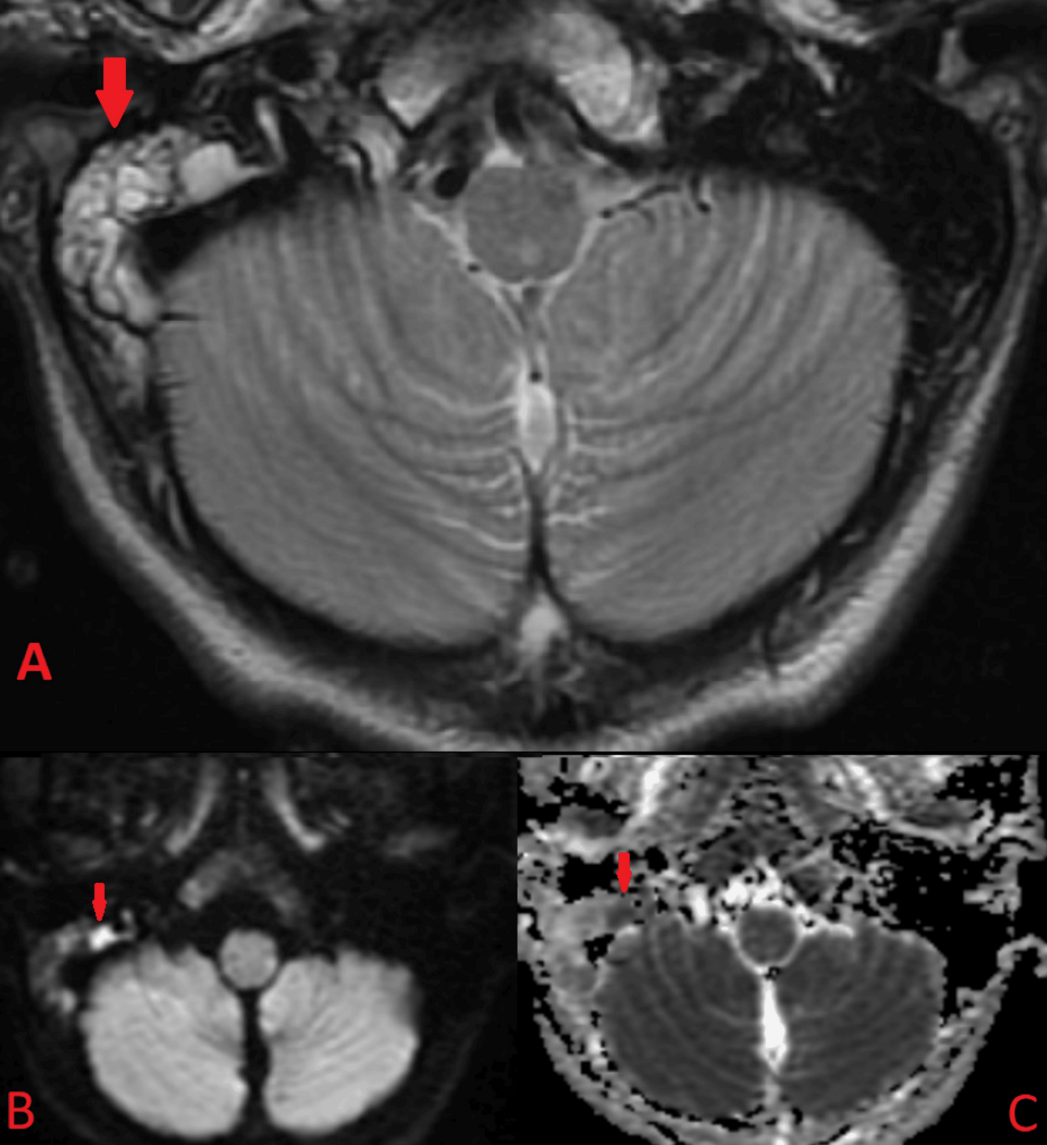
MRI brain axial T2 fast spin echo (A), axial DWI (B), and axial ADC (C) at the level of the mastoid air cells and posterior fossa. There is an increased T2 signal within the right mastoid air cells (red arrows) consistent with complete opacification. On corresponding DWI and ADC, there is diffusion restriction likely secondary to infection/pus. This is highly suggestive of mastoiditis in the correct clinical setting.

**Figure 3:**
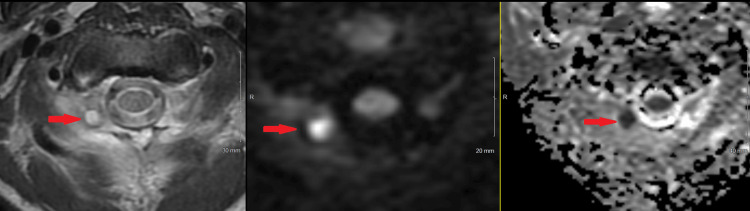
MRI cervical spine axial T2 fast spin echo (left), axial DWI (center), and axial ADC (right) sequences. Findings include a well-circumscribed T2 hyperintense fluid collection involving the right C1-C2 facet joint with diffusion restriction consistent with abscess.

**Figure 4:**
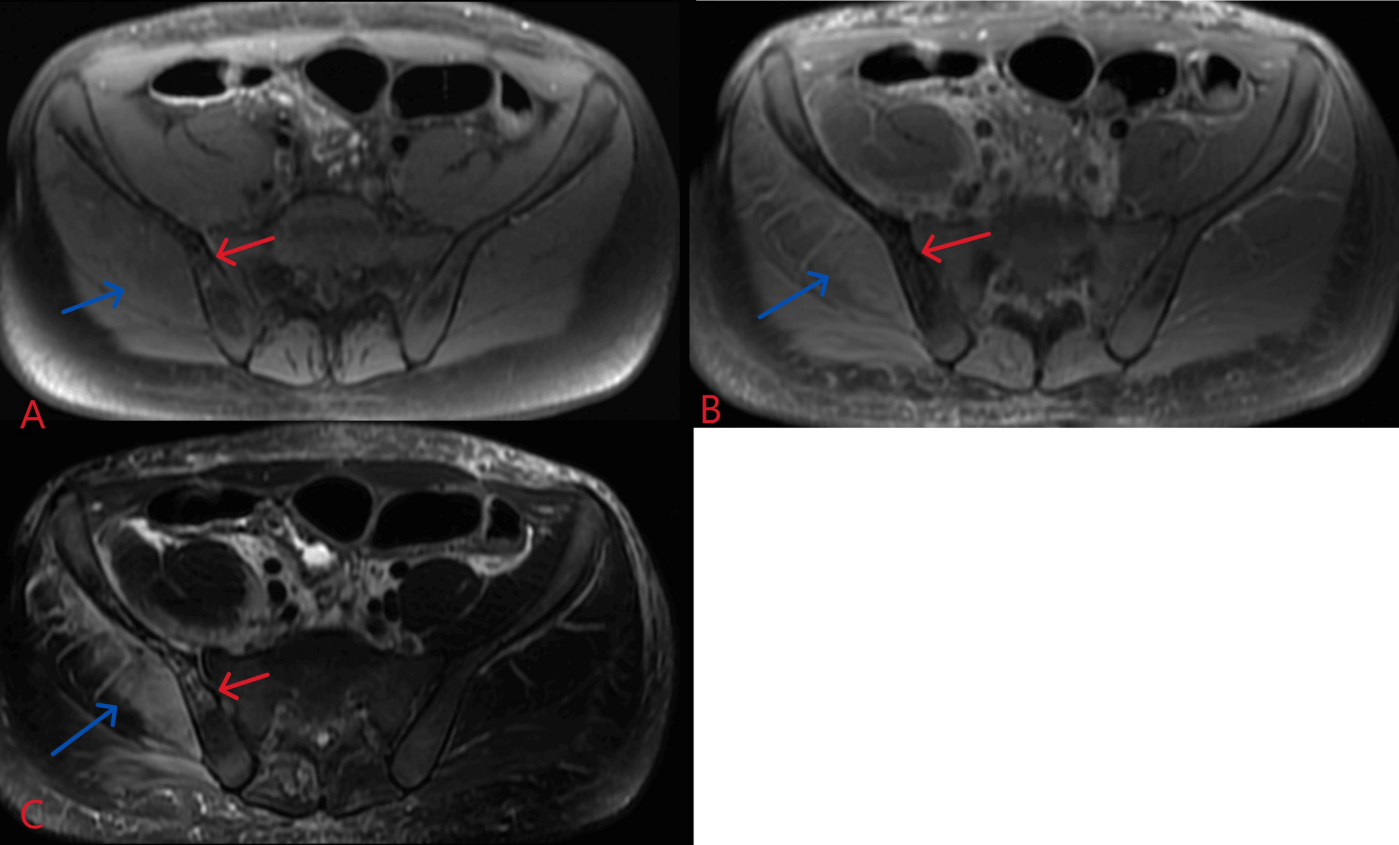
Initial MRI pelvis. T1 pre-contrast (A), T1 post-contrast (B), and T2 (C). There is a diffusely increased T2 signal in the muscles of the right hip (blue arrows), including the gluteal musculature, and the iliopsoas, which are enlarged and less well-defined relative to the contralateral musculature consistent with inflammatory changes/myositis. There is also an increased T2 signal of the subcutaneous tissues about the right hip. No discrete fluid collection is appreciated. Heterogeneous T2 hyperintense and T1 hypointense signal abnormality is visualized extending throughout the right iliac bone (red arrows). This corresponds to a region of decreased enhancement on the postcontrast sequence consistent with osteomyelitis with osteonecrosis.

**Figure 5:**
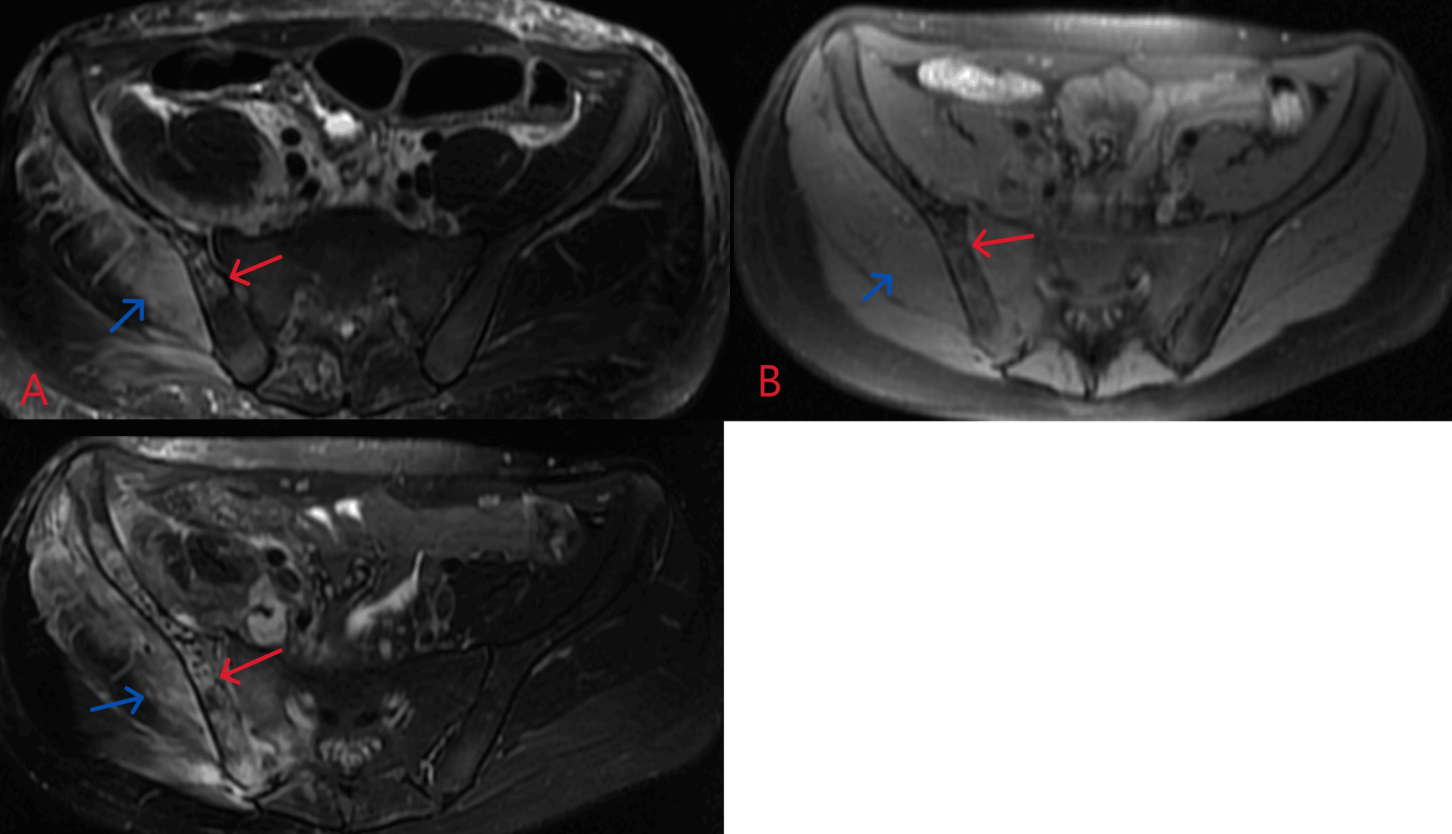
Follow-up MRI pelvis two weeks after initial presentation. T1 pre-contrast (A), T1 post-contrast (B), and T2 (C). Within the right iliac bone, there is a progression of the previously seen heterogeneous decreased T1 signal, increased T2 signal, and lack of postcontrast enhancement within the iliac bone consistent with advanced osteomyelitis with osteonecrosis (red arrows). There is similar myositis throughout the musculature of the right hemipelvis and hip region (blue arrows).

Initial imaging revealed right otitis media and mastoiditis (Figures 1-2), septic arthritis of C1-C2 (Figure 3), and osteomyelitis with osteonecrosis of the iliac bone, as well as diffuse myositis and cellulitis in the right hip and hemipelvic region (Figure 4). A follow-up MRI of the right pelvis two weeks after presentation further demonstrated advanced osteomyelitis with osteonecrosis involving the right iliac bone, osteomyelitis involving the right upper sacrum and right sacroiliac (SI) joint, and myositis with the development of a small abscess in the superolateral right gluteus medius (Figure 5). Subsequent image-guided incision and drainage (I&D) procedures were performed based on the MRI findings.

Negative cultures resulted from the hip region cultures obtained during washout. Therefore, samples were sent for polymerase chain reaction (PCR) analysis, yielding the presence of F. necrophorum, leading to the initiation of targeted antibiotic therapy. Initially, the patient was started on meropenem, but due to a down-trending absolute neutrophil count (ANC) and transaminitis requiring multiple dose adjustments, the antibiotic regimen was modified to ceftriaxone and metronidazole.

The patient required multiple washouts and debridements, intravenous antibiotics, and pain control. Follow-up MRI studies were crucial in monitoring the progression of the disease, guiding surgical decision-making, and assessing treatment response. Inpatient work-up was negative, and the patient was discharged with outpatient follow-up to assess for any signs of treatment failure or recurrence, for instance, demonstrated by symptomatology, physical exam, wound healing, and elevations in white blood cell counts and acute phase reactants.

## Discussion

F. necrophorum is a gram-negative anaerobic bacterium commonly found in the normal flora of the oropharynx. While it is implicated in infections within the head and neck region, and is a recognized causative agent of conditions, such as peritonsillar abscesses [1], Lemierre's syndrome [2], and deep neck space infections [3], the rarity in which of F. necrophorum is demonstrated in metastases to the hip and pelvis suggests that the pathogen may not be immediately suspected, potentially leading to delays in proper treatment due to challenges in diagnosis and therapy. While evaluation with imaging is an important step, the presence of this pathogen was found with PCR over standard culturing techniques.

The migration of F. necrophorum from its primary habitat in the oropharynx to distant sites such as the hip bone is an unusual occurrence. The mechanisms underlying this dissemination remain poorly understood but may involve hematogenous spread with risk factors, including disruption of mucous barriers or other tissue injury. Osteomyelitis or prolonged mastoiditis with F. necrophorum prompts investigation for immunodeficiency syndromes, which our patient was worked up for in the hospital and will be further evaluated as an outpatient [7].

Radiological imaging, especially MRI, plays a vital role in characterizing the extent of the disease and guiding surgical interventions [6]. In this case, MRI helped identify the involvement of the right iliac bone, sacrum, SI joint, and soft tissue, which facilitated prompt treatment decisions. The confirmation of F. necrophorum through tissue sampling and PCR highlighted the specific pathogen responsible for the infection and allowed for the optimization of antibiotic therapy.

## Conclusions

This case report sheds light on the atypical presentation of F. necrophorum osteomyelitis in the hip region of a pediatric patient. F. necrophorum, primarily associated with head and neck flora, rarely manifests as osteomyelitis in distant sites. The unique nature of this case highlights the need for a high index of suspicion and careful diagnostic evaluation when encountering osteomyelitis, especially in cases where clinical and radiological findings deviate from the expected.

In conclusion, the comprehensive management of F. necrophorum-related infections requires a multidisciplinary approach, including collaboration between radiologists, pediatricians, surgeons, and infectious disease specialists. This case underscores the importance of considering uncommon pathogens and the potential for atypical presentations in the assessment and treatment of pediatric patients with osteomyelitis.
